# Preconceptional Counseling in Women with Hyperthyroidism

**DOI:** 10.3390/medicina60020234

**Published:** 2024-01-29

**Authors:** Luminita Nicoleta Cima, Mihaela Tarna, Carmen Sorina Martin, Anca Elena Sirbu, Iulia Soare, Anca Maria Panaitescu, Nicolae Gica, Carmen Gabriela Barbu, Simona Fica

**Affiliations:** 1 Department of Endocrinology and Diabetes, Nutrition and Metabolic Diseases, “Elias” Emergency University Hospital, 011461 Bucharest, Romania; 2 Department of Obstetrics and Gynecology, Carol Davila University of Medicine and Pharmacy, 050474 Bucharest, Romania; 3 “Filantropia” Clinical Hospital, 011171 Bucharest, Romania

**Keywords:** thyrotoxicosis, hyperthyroidism, thyroid disease, Graves’ disease, gestational transient thyrotoxicosis, pregnancy, preconception counseling, antithyroid drugs, congenital anomaly

## Abstract

Preconception evaluation of couples wishing to conceive is an important step toward a healthy pregnancy and it is especially important in people with a chronic condition or at genetic risk. The most common endocrine disorders in women at reproductive age are those involving the thyroid gland and it is well recognized that hyperthyroidism (HT), over-function of the thyroid gland, is associated with risks of maternal, fetal, and neonatal complications. The aim of this paper is to review the latest evidence regarding the components of preconception counseling in women with HT that contemplate a pregnancy. We also want to raise awareness among healthcare professionals about the importance of periconceptional counseling in improving pregnancy outcomes and avoid maternal and fetal complications related to thyroid dysfunction. In women with Graves’ disease seeking pregnancy, it is essential to discuss all the treatment options along with the associated risks and benefits. Extensive prospective studies are still needed to understand the implications of current recommended strategies for the management of HT in preconception and during pregnancy.

## 1. Introduction

Thyroid dysfunction is the most common endocrine disorder in women at reproductive age. Thyroid pathology might interfere with pregnancy outcomes; therefore, all women with thyroid disorders that are planning for a pregnancy should, ideally, receive preconceptional counseling to review their thyroid function [1,2,3].

It has been shown by previous studies that hyperthyroidism can be found in about 0.2–2.7% of all pregnancies [4,5]. Also, it is well recognized that hyperthyroidism (HT) is associated with risks of maternal, fetal, and neonatal complications, including miscarriages, stillbirths, neuro-intellectual impairment of the offspring, neonatal hyperthyroidism, and low birth weight [6,7]. Central hypothyroidism may develop as a consequence of an excess of thyroid hormones, impairing the maturation of the fetal hypothalamic–pituitary–thyroid axis [8]. Furthermore, observations from a large number of studies indicate that antithyroid drugs (ATDs) have the potential to be teratogenic and may cause fetal or neonatal hypothyroidism with important consequences for the neurocognitive development of the newborn [9,10,11,12].

Therefore, one of the main purposes of preconception counseling in these situations is to optimize thyroid function before achieving any pregnancy. All couples should be advised to use contraception until an euthyroid state is achieved in the mother. The doctor should discuss with the future parents the effects that thyroid disease might have on pregnancy and the fetus and the influence that pregnancy might have on thyroid disease [1]. Also, the potential maternal and fetal side effects of all available treatment options should be reviewed.

Thyrotoxicosis is relatively common among women of childbearing age and in approximately 90% of cases is caused by Graves’ disease (GD) [13]. Also, many pregnant women are first diagnosed with GD during early pregnancy [2]. Other more rare causes of thyrotoxicosis occurring in pregnancy are toxic adenoma, multinodular goiter, thyroiditis, and gestational transient thyrotoxicosis (GGT), the latter typically reported in the first trimester in pregnancies that present with hyperemesis gravidarum in relation to high circulating concentrations of human chorionic gonadotropin (HCG) and subsiding by 14–18 weeks of gestation [8,14,15]. Therefore, in patients presenting with thyrotoxicosis in early pregnancy, it is important to undertake a differential diagnostic approach in order to detect the underlying etiology, offer the appropriate treatment, and avoid undesired fetal side effects [16].

Preconception evaluation of couples wishing to conceive is an important step toward a healthy pregnancy and it is especially important in people with a chronic condition or at genetic risk. For reproductive-age women with hyperthyroidism desiring pregnancy, preconception counseling should always be offered and given considering the physiologic adaptations that occur during pregnancy and the importance that thyroid hormones have in normal fetal development. Preconception counseling should involve, ideally, a multidisciplinary team to optimize the outcomes for both the mother and her fetus [1,17].

The aim of this paper is to review the latest evidence regarding the components of preconception counseling in women with hyperthyroidism that contemplate a pregnancy. We also want to raise awareness among healthcare professionals about the importance of periconceptional counseling to improve pregnancy outcomes and avoid maternal and fetal complications.

## 2. Methods

We conducted a literature review of international online databases, including PubMed, Google Scholar, and Cochrane, for relevant information regarding the screening, diagnosis, and treatment of thyrotoxicosis during the preconception period and its impact on fertility, pregnancy, and the newborn using a combination of the keywords “thyrotoxicosis”, “hyperthyroidism”, “thyroid disease”, “Graves’ disease”, “gestational transient thyrotoxicosis”, “pregnancy”, “preconception counseling”, “ATD”, “MMI”, “CMZ”, “PTU”, and “congenital anomaly”. Additional publications were obtained from references cited in individual articles. The search was conducted by two authors (C.L.M. and T.M.) with discrepancies resolved by consensus. We prioritized the latest clinical practice guidelines, most-cited reviews, and meta-analyses.

## 3. Results

### 3.1. Screening for Thyroid Disease in Preconception

Specific screening for thyroid dysfunction before pregnancy is still a matter of debate. To justify universal screening, there are several requirements that need to be fulfilled: a condition must be highly prevalent and have adverse health outcomes, there should be an effective treatment, and the approach should be cost-efficient. Thyroid dysfunction fulfills almost all of these prerequisites; however, in order to be able to formulate evidence-based recommendations, further research is still needed.

The American Thyroid Association (ATA) and the European Thyroid Association (ETA) guidelines recommend verbal screening and a clinical evaluation for all patients presenting for preconceptional visits and for all newly pregnant women in order to identify women at risk of thyroid disease (those aged >30 years, those with a medical history of thyroid dysfunction/thyroid surgery/head or neck radiation or radioactive iodine therapy/pregnancy loss/preterm delivery/infertility/autoimmune disorders/severe obesity/multiple prior pregnancies, those with a family history of thyroid dysfunction/autoimmune thyroid disease or signs or symptoms of thyroid dysfunction, those with a goiter, those who use amiodarone/lithium/an iodinated radiologic contrast, or those who reside in a geographical area with moderate/severe iodine insufficiency (Table 1) [2,3]. Nevertheless, a study from China that enrolled almost 3000 pregnant women demonstrated that a case-finding strategy for screening thyroid function in the high-risk group would miss about 81.6% of pregnant women with hypothyroidism and 80.4% of pregnant women with hyperthyroidism [5].

The most frequently utilized tests for the assessment of thyroid status in both pregnant and non-pregnant patients include TSH, FT4, TT4, and TPOAb from the blood, and they are relatively cheap to perform and readily available. During pregnancy, especially in the first trimester, changes in thyroid hormone levels that are indicative of hyperthyroidism must be distinguished from physiological changes due to the action of HCG on the TSH receptor with an increase in thyroid hormone production and, consequently, a suppression in the TSH level [8]. In cases where there is doubt about the underlying etiology, TSH receptor antibody (TRAb) levels can help distinguish between GGT and Graves’ disease. However, in pregnancy, the results are not always irrefutable because of the characteristic immunosuppression [18].

### 3.2. Graves’ Disease and a Future Pregnancy

#### 3.2.1. Epidemiology/Definition/Diagnostics

GD is an autoimmune disease characterized by the presence of TSH receptor antibodies (TRAbs), usually causing hyperthyroidism (high FT4 or FT3, with suppressed TSH), goiters, and nonthyroid manifestations, such as Graves’ orbitopathy or dermopathy [19,20]. Inhibitory or neutral TRAbs have also been described.

The peak incidence of GD is in women of age 30–50, and, therefore, it often presents during the preconception period, affecting 0.4–1.0% of fertile females, during pregnancy, affecting about 1 in 500 pregnant women, or during the postpartum period [19,20,21].

Women of reproductive age with GD are encouraged to discuss pregnancy planning or contraception with their general physician, an endocrinologist, or a specialized obstetrician at every possible occasion. It is important to plan ahead in order to have the best possible settings for a pregnancy in these patients. Preconception counseling will address the following issues: the effects of GD on fertility, pregnancy, and fetal and newborn outcomes, the effects of pregnancy on the evolution of GD, treatment options and their effect on a future pregnancy, medication adequacy and requirements, the need for a specialized follow-up during a future pregnancy, breastfeeding, and the post-partum period.

#### 3.2.2. Effects of Graves’ Disease on Fertility, Pregnancy, and Fetal/Newborn Outcomes

Untreated and uncontrolled Graves’ disease can transiently affect fertility and the ability to conceive and carry a pregnancy [22]. In patients with GD, adequate control of the thyroid status should be first achieved before addressing conception. Contraception is recommended until the euthyroid state is reached.

Physicians attending to women that have or have had GD should be aware that TRAbs can cross the placenta during pregnancy by employing the physiological IgG transplacental transport mechanisms and act on the fetal thyroid gland. TRAbs should be measured at the time of presentation in all pregnant women or women seeking pregnancy in the near future who have a history of Graves’ hyperthyroidism with or without ATD therapy, after radioiodine ablation (RAI) or surgery, or who have a history of fetal/neonatal thyroid dysfunction [23].

Pregnancy-related GD complications are associated with poorly controlled hyperthyroidism, which can have severe consequences for the mother and the baby. These adverse effects of moderate to severe hyperthyroidism include, on the one hand, maternal and obstetrical complications (miscarriage, gestational hypertension, preeclampsia, placental abruption, congestive heart failure, thyroid storm, premature delivery, premature rupture of a membrane) [8] and, on the other hand, disease-related congenital malformation, hyperthyroidism, central hypothyroidism, goiter, hip dysplasia, congestive heart failure, intrauterine growth restriction, low birth weight, prematurity, respiratory or neonatal intensive care unit treatment, neurologic and neurobehavioral disorders, and treatment-associated fetal and neonatal complications [7,24,25,26]. A recent study performed on 3839 mother–child pairs for IQ, with magnetic resonance imaging (MRI) scans being available from 646 children, demonstrated that both low and high maternal free thyroxine concentrations during pregnancy were associated with lower child IQ and lower grey matter and cortex volume [9]. This association between high maternal free thyroxine and low child IQ may raise concerns regarding the approach of ATD withdrawal in pregnant GD women with prior control of the disease before pregnancy that might carry a potential risk of adverse effects on child neurodevelopment outcomes, but further research is needed. In addition, ATD medication can cause fetal and neonatal hypothyroidism and goiter [19,23,27].

No association has been found between subclinical hyperthyroidism and pregnancy complications [27].

**Figure 1:**
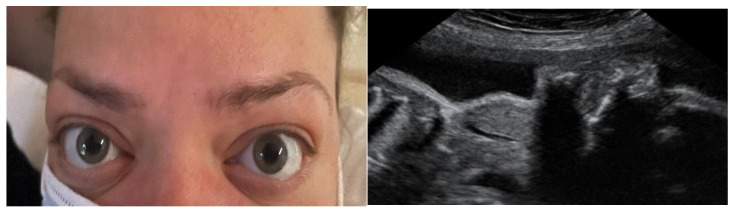
Orbithopathy in a pregnant woman with Graves’ disease (**left**). The fetus was diagnosed with goiter by ultrasound (**right**).

Neonatal hyperthyroidism is found in 1–5% of babies delivered by mothers with a history of Graves’ disease (Figure 1). There is also a potential risk of hyperthyroidism in the fetus and neonate even in those cases where the mother undertook thyroidectomy or ablation [17,28], and this risk is explained by residual TRAb levels. TRAb titers correlate with GD activity and the probability of relapse and predict the risk of fetal and neonatal involvement [23]. If, at the initial evaluation before or in early pregnancy, the TRAb level is <2.5–3 times the upper limit of the normal range, the measurement does not need to be repeated; however, if elevated values are found (≥2.5–3) it should be repeated at 18–22 weeks of gestation. Maternal TRAb titers ≥2.5–3 times the upper limit of the normal range in the second or third trimester predict fetal and neonatal hyperthyroidism risk [19].

#### 3.2.3. Treatment for GD and Implications for a Future Pregnancy: Who and How to Treat

When women are diagnosed with Graves’ disease prior to pregnancy, it is essential for them to receive all the information regarding the risks and benefits of all the treatment alternatives. Treatment options to control hyperthyroidism in GD include the ATDs (propylthiouracil (PTU) and methimazole (MMI)), RAI, and thyroidectomy. The couple’s plans for pregnancy and lactation have important implications for the choice of treatment [19].

Pregnancy should be postponed until a stable euthyroid state is reached (two sets of normal thyroid function tests at least 1 month apart with no change in therapy between tests) [2,8].

The goal of the treatment is to achieve and maintain a mild state of maternal hyperthyroidism, with fT4 near the upper limit of the normal range, to prevent fetal hypothyroidism [2,8,17].

**a.**  **Antithyroid drugs**

Women currently on ATDs should use contraceptive methods, receive counseling in case of a missed period, and immediately perform a pregnancy test and contact their caregiver. Both MMI and PTU are able to cross the placental barrier. It has long been known that MMI is teratogenic, but it has only recently been clarified that PTU is also associated with some birth defects [11,23,29,30,31]. The teratogenic effect of ATDs is most remarkable when used during the first trimester (organogenesis) [19,29,32]. Conflicting data from observational studies have made it necessary to compile several recent meta-analyses that analyzed the risk ratios for congenital anomalies after MMI/CMZ and/or PTU exposure during pregnancy (Table 2 and Table 3). Furthermore, it is considered that hyperthyroidism in itself might have teratogenic potential independent of drug exposure [32].

The birth defects associated with the two drugs are different. MMI-associated birth defects are typically more severe and include aplasia cutis, choanal atresia, esophageal atresia, abdominal wall defects, and cardiac defects like ventricular septal defects [11,34]. In addition, the Italian Drug Agency recently issued a warning on the risk of acute pancreatitis related to MMI use [37]. Regarding PTU use in pregnancy, possible defects in the fetus associated with its use are cysts of the face and neck and, in male fetuses, urinary abnormalities. PTU has also been associated with cases of fulminant hepatic failure that have also been reported in pregnancy. Therefore, because of all these factors, PTU is no longer the drug of choice during pregnancy, except for early pregnancy [19,23,29,38]. Moreover, a recent cohort study based on data from the Swedish nationwide registry, which includes 684,340 children live-born in Sweden from 2006 to 2012, showed that the cumulative incidence of birth defects is similar in children exposed to MMI (6.8%, *p* = 0.6) or to PTU (6.4%, *p* = 0.4) vs. those non-exposed (8.0%) [39]. It is also noteworthy that these results were endorsed by a recent meta-analysis that included data from 13 randomized controlled trials (RCTs) and cohort studies including 18,948 infants born from hyperthyroid mothers treated with PTU. The study confirmed the efficacy of PTU in the treatment of hyperthyroid pregnant women. In addition, the authors concluded that the risks of adverse pregnancy outcomes were not increased [36].

In concordance with the recommendations from the most recent international guidelines, the findings of this recent meta-analysis support using PTU in pregnancy complicated by hyperthyroidism to improve clinical outcomes. Therefore, when the continuation of medical therapy is implied, it is advisable to change from MMI to PTU before conception or immediately after stopping contraception in healthy young women with regular menstrual cycles that can rapidly achieve a pregnancy [8,19]. A strong argument is that most women discover that they are pregnant after the gestational age of 6–10 weeks when there is the highest risk of congenital malformation associated with ATDs. A reasonable recommendation is for PTU to be used in the first trimester and for MMI to be used in a later stage of pregnancy if necessary [38]. Shifting from MMI to PTU is not easy, and clinicians should know that it might take some time to obtain a good degree of control over the thyroid function. A dose ratio of approximately 1:20 (MMI:PTU) should be used (e.g., MMI 5 mg/d = PTU 50 mg twice daily) [2].

Another concerning issue was underlined by a recent meta-analysis performed by Agrawal et al. that showed an increased risk for congenital anomalies in hyperthyroid patients (in those exposed either to CMZ/MMI or PTU) that switched between ATDs in pregnancy (RR, 1.51; 95% CI, 1.14–1.99). Unfortunately, it is difficult to infer a conclusion on this topic because the timing of the ATD switch varied between studies, with some cases undertaken before pregnancy [33]. Thus, according to current clinical practice guidelines, changing from CMZ/MMI to PTU at conception does not seem to reduce the risk for congenital anomalies; on the contrary, exposure to both drugs may in fact be associated with a higher risk (Table 2, Table 3 and Table 4). The results of this recent meta-analysis are in concordance with the previous results of a meta-analysis that investigated the risks of congenital defects in pregnant patients exposed to both PTU and MMI [24,32,34,35].

Therefore, the debate on the management of hyperthyroid patients in pregnancy remains open. Conducting an adequately powered RCT with a direct comparison between drugs would potentially solve this dilemma; however, this is unlikely to be feasible in this population given the potential ethical dilemmas and the large population and amount of time needed. Therefore, meta-analysis of observational studies represents, probably, the next best level of evidence for evaluating the safe use of ATDs in pregnancy [33]. Also, we should consider the fact that hyperthyroidism by itself may have teratogenic potential independent of ATD exposure as suggested by some authors [32] and this can not be quantified by itself.

The patient must also be informed about the necessity of frequent blood tests during pregnancy in order to monitor thyroid hormone levels and adjust the ATD dosage [40]. ATDs are also able to be transferred in small amounts into breast milk, but low doses are safe during lactation [8]. However, when high doses of ATDs are necessary to control hyperthyroidism in females with GD, ablative therapy should be considered before conception [23].

A recent Japanese study that included 283 pregnant women with GD whose treatment was changed from MMI to potassium iodide (KI) in the first trimester (iodine group) and 1333 patients treated with MMI alone (MMI group) demonstrated a significantly lower incidence of major anomalies 4/260 (1.53%) in the iodine group, compared with 47/1134 (4.14%) in the MMI group. Also, there were no cases of thyroid dysfunction or goiter in the neonates exposed to KI. The authors suggested that, in their population, in an iodine-sufficient region, changing to KI from MMI in hyperthyroid GD patients during the first trimester of pregnancy may reduce the incidence of congenital defects [41], but this hypothesis needs to be tested in a larger and more diverse population.

**b.**  **Definitive therapy with thyroidectomy**

Thyroidectomy is the most effective therapy for decreasing TRAb levels [23]. Therefore, patients who have very high, persistent TRAb titers may be the best candidates for surgery [40]. Following thyroidectomy, conception should be postponed until TSH levels are optimal on LT4 therapy and a stable euthyroid state has been achieved [8,19]. Nevertheless, the patient should be informed about the risk for fetal and neonatal hyperthyroidism that may occur even if the mother has previously undergone a thyroidectomy [17,28].

Although not practical and not usually performed, thyroidectomy can be undertaken in pregnancy, preferably in the second trimester, if required [1].

**c.**  **Definitive therapy with radioactive iodine (I131)**

Radioactive ablative with iodine (RAI) therapy is not a recommended choice for hyperthyroid women who desire pregnancy in the near future for multiple reasons. First, a pregnancy test should be performed before ablation given its absolute contraindication in pregnant women. In addition, patients should be advised to use contraception for at least 6 months after radioactive iodine treatment in order to achieve the optimum thyroid hormone replacement level [40]. Particularly, a subset of young patients with severe GD may not be able to reach a stable euthyroid state within the first year after RAI therapy, which could delay the moment of conception [2,42,43,44,45].

RAI treatment implies a transient rise in the TRAb titer above baseline levels, which usually reaches its highest point at three months and persists for up to a year, and this could lead to an increased risk for fetal or neonatal hyperthyroidism [19]. Consequently, patients with high TRAb titers or severe hyperthyroidism may be more suitable for other therapeutic options, such as surgery [46].

**d.**  **Conservative management**

A conservative approach could be useful for patients with subclinical or even overt hyperthyroidism, if it is mild and asymptomatic, who have subnormal TSH and free T4 levels above the reference range or total T4 and T3 levels >1.5 times the upper limit of the normal range for nonpregnant patients [8].

According to international guidelines, in carefully selected women who are euthyroid on low-dose ATDs before pregnancy (MMI ≤ 5–10 mg/day or PTU ≤ 100–200 mg/day), the physician may consider discontinuing all antithyroid medication. The decision to stop medication should take into account the disease history and must be encouraged in the case of a minimum duration of therapy of 6 months, the absence of goiter or a small goiter size, adequate thyroid function, and no positive TRAb titers. Close monitoring of symptoms should occur and thyroid function tests should be performed every 1–2 weeks, with an extension to 2–4 weeks during the second and third trimesters, if the pregnant woman remains clinically and biochemically euthyroid. In these cases, severe hyperthyroidism must be avoided [2,19].

This approach is based on the concept that the recurrence of hyperthyroidism usually does not happen for several weeks; therefore, the ATD can be reinstated after the first trimester. However, although this strategy has been empirically promoted in recent guidelines, it requires further research and evidence [19].

**e.**  **Beta-adrenergic blocking agents**

β-blockers may be used to control hypermetabolic symptoms, in the absence of contraindications, until patients have become euthyroid on ATDs or after RAI therapy or surgery. Initial doses used are 10–40 mg every 6–8 h for propranolol and 25 to 50 mg/daily for metoprolol. Doses should be reduced as clinically indicated, and they can usually be discontinued in 2–6 weeks. It must be noted that long-term treatment with β-blockers, especially atenolol, has been associated with adverse effects, including intrauterine growth restriction, fetal and neonatal bradycardia, and neonatal hypoglycemia; therefore, it should be avoided for more than 2–6 weeks during pregnancy [13]. It has also been emphasized that there may be a higher rate of spontaneous pregnancy loss in patients treated with both ATDs and β-blockers compared with patients receiving only MMI [47] (Table 5).

#### 3.2.4. Effects of Pregnancy on the Evolution of GD

Graves’ disease, like many other autoimmune conditions, can improve during pregnancy; however, several studies have reported flares and exacerbations post-partum [49,50]. It is important, therefore, to counsel women to check their thyroid status in the months following delivery. There is no evidence on the question of whether pregnancy alters the long-term evolution of GD.

### 3.3. Autonomously Functioning Nodules/Toxic Nodular Goiter

#### 3.3.1. Epidemiology/Definition/Diagnostics

Toxic nodular goiter usually occurs later in life, being a rare cause of hyperthyroidism in women of childbearing age. The onset of hyperthyroidism in this setting is typically gradual and less severe than in GD, and TRAbs are absent. A definitive diagnosis of nodular autonomy demands a radioactive iodine uptake test, which is contraindicated in pregnancy [19].

#### 3.3.2. Effects on Pregnancy and Fetal/Newborn Outcomes

Adverse outcomes are similar to those mentioned in GD, except for the fact that the use of ATDs in pregnancy leads to a higher risk for fetal hypothyroidism since, unlike in GD, the fetus is not exposed to maternal thyroid-stimulating antibodies that can counteract the effects of antithyroid drugs on the fetal thyroid [19]. Furthermore, the natural history of overt hyperthyroidism associated with autonomously functioning nodules is progressively worsening hyperthyroidism with low rates of remission.

#### 3.3.3. Who and How to Treat

In light of the natural history of the disease and the increased risk for fetal hypothyroidism secondary to ATDs in pregnancy, definitive therapy with thyroidectomy or RAI should be strongly recommended before pregnancy [19].

Likewise, for GD, in toxic adenoma or toxic multinodular goiter, a conservative strategy could be useful in patients with subclinical or overt hyperthyroidism (if mild and asymptomatic) considering the physiological increase in thyroid hormone needs in pregnancy that may ameliorate hyperthyroidism [8]. On the other hand, the plasma levels of hCG are the highest toward the end of the first trimester and may theoretically increase hormone production in the normal thyroid tissue and aggravate hyperthyroidism, making a close follow-up necessary. Additionally, hormone production in autonomous thyroid nodules is dependent on the available amount of iodine. Therefore, these patients should probably avoid iodine-containing supplements during pregnancy [2].

## 4. Discussion

Normal thyroid secretion is essential for successful conception and normal fetal development. As has been shown previously, overt thyrotoxicosis during pregnancy is associated with adverse outcomes for both the mother and fetus. Therefore, it should be diagnosed, treated, and ideally cured before gestation, but this may not always be possible.

Evidence of increased serum TRAb levels, the presence of goiter, an increase in the T3 level or T3/T4 ratio, and signs of orbitopathy on clinical examination are characteristics of GD and differentiate it from GTT [2,8,15]. GTT is usually associated with mild thyrotoxic symptoms, which spontaneously subside by 14–18 weeks of gestation as HCG values decrease and do not require treatment [8,15].

Treatment objectives in pregnant women with hyperthyroidism are to provide an excellent fetal and maternal outcome by preventing the complications induced by uncontrolled maternal hyperthyroidism as well as preventing the development of fetal hypothyroidism [2,3].

Therapeutic strategies depend on the etiology and severity of the disease and the timing of the diagnosis in relation to pregnancy (before or during early or advanced pregnancy) and should take into account the risk of side effects.

Most women with hyperthyroidism presenting for preconception counseling have a confirmed diagnosis of GD. As a general rule, the pregnancy should be postponed and adequate contraceptive methods must be used until a euthyroid state is achieved, demonstrated by at least two normal thyroid function tests 4 weeks apart on the same medication [1,2]. The lowest doses of ATDs should be used to maintain a serum FT4 level at or just above the upper reference limit, thus avoiding fetal hyper/hypothyroidism and minimizing the risk of ATD-induced teratogenic effects. In women with GD treated for a long period with ATDs who are well controlled on low ATD doses, medication may be withdrawn during early pregnancy in order to prevent teratogenic effects in the fetus [2,19].

Frequent TSH and FT4 monitoring is needed in hyperthyroid women to obtain a good degree of control of the thyroid function during pregnancy and after delivery, particularly in GD patients, in whom recurrence or exacerbation of hyperthyroidism occurs in a high percentage of cases [2,8,19].

There is insufficient evidence to make a clear recommendation on iodine supplementation in women with a history of Graves’ hyperthyroidism or in women with ATD-treated hyperthyroidism. Further studies are required to clarify this issue.

Further high-quality research including a larger and more diverse population is necessary regarding the conservative management of mild hyperthyroidism in Graves’ disease, autonomously functioning nodules and toxic nodular goiter, and the potential use of KI for the control of hyperthyroidism.

Limitations to our knowledge regarding this subject include a lack of consensus regarding the decision to recommend universal thyroid disease screening in preconception or during pregnancy for all women. This subject remains under debate and there is no clear evidence that treatment has a positive effect on pregnancy outcomes [2]. Nevertheless, a large percentage of general practitioners, obstetricians, and gynecologists perform screening procedures in women of reproductive age.

Extensive prospective studies are still needed to evaluate the currently recommended management strategies in women with hyperthyroidism during pregnancy. We recommend a multidisciplinary approach that comprises at least an endocrinologist, an obstetrician, and a neonatologist in order to reduce fetal, neonatal, and maternal risks in hyperthyroid women.

## 5. Conclusions

The current data available are not consistent enough to provide evidence-based recommendations regarding different thyroid diseases before or during pregnancy. The current recommendations according to the most recent data available on periconceptional counseling in women of reproductive age with hyperthyroidism can be synthesized as follows:

− Screening for thyroid disease is recommended only in the presence of risk factors;
− In women with GD seeking pregnancy, it is essential to discuss all the treatment options along with the associated risks and benefits. ATDs might seem a comfortable option, but they come with important fetal risks. RAI ablation might interfere with fertility and the time of conception, while thyroidectomy seems to be the most effective at decreasing TRAb levels. A conservative strategy might be an option for carefully selected patients;
− In women with autonomously functioning nodules or toxic multinodular goiter, definitive therapy with thyroidectomy or RAI should be strongly recommended before pregnancy because the high levels of hCG present in early pregnancy may increase hormone production in the normal thyroid tissue and aggravate hyperthyroidism. The use of ATDs in these patients during pregnancy is associated with a higher risk for fetal hypothyroidism since, unlike in GD, the fetus is not exposed to maternal thyroid-stimulating antibodies that can counteract the effects of ATD on the fetal thyroid.

## Figures and Tables

**Table 1:** Categories of patients that could benefit from screening for thyroid function before conception. Verbal screening and clinical evaluation are recommended for all patients before planning a pregnancy.

Categories of Patients
Age > 30 years
Medical history of thyroid dysfunction
Thyroid surgery
Head or neck radiation or radioactive iodine therapy
Autoimmune disorders
Family history of thyroid dysfunction
Goiter
Use of amiodarone or lithium
Recent exposure to an iodinated radiologic contrast
Residence in an area of moderate/severe iodine insufficiency
Recurrent pregnancy loss
Preterm delivery
Previous stillbirth
Infertility
Multiple prior pregnancies
Diabetes

**Table 2:** Meta-analysis of risk ratios for congenital anomalies after MMI/CMZ exposure during pregnancy.

Author, Year	No. of Studies Included	MMI/CMZ	Control	Crude RR, 95% CI for Congenital Anomalies	Adjusted RR, 95% CI for Congenital Anomalies	No. of Anomalies/1000 Live Births
		Events/Total	Events/Total			
Agrawal et al., 2022 [33]	7	269/3521	340,801/5,364,080	RR 1.37; 95% CI 1.22–1.54	RR 1.28; 95% CI 1.06–1.54	17.2
Morales et al., 2021 [32]	4				RR 1.28; 95% CI, 1.06–1.54	17.8
Song et al., 2017 [34]	4	108/1395	83,426/1,461,088	RR 1.61; 95% CI 1.32–1.97		
Li, H. et al., 2015 [24]	7	170/2993	83,490/1,463,996	RR 1.64; 95% CI 1.39–1.92		
Li, X. et al., 2015 [35]	6	159/2662	83,450/1,462,586	RR 1.76; 95% CI 1.47–2.10		

**Table 3:** Meta-analysis of risk ratios for congenital anomalies after PTU exposure during pregnancy.

Author, Year	No. of Studies Included	PTU	Control	Crude RR, 95% CI for Congenital Anomalies	Adjusted RR, 95% CI for Congenital Anomalies	No. of Anomalies/1000 Live Births
		Events/Total	Events/Total			
Miao et al., 2022 [36]	11	143/4459	483/14,001	RR 1.03; 95% CI, 0.84–1.25		
Agrawal et al., 2022 [33]	6	859/12,662	340,811/5,363,707	RR 1.18; 95% CI, 1.11–1.26	RR 1.16; 95% CI, 1.08–1.25	9.8
Morales et al., 2021 [32]	4				RR 1.16; 95% CI 1.08–1.25	10.2
Song et al., 2017 [34]	4	117/2189	83,459/1,461,804	RR 1.29; 95% CI 1.07–1.55		
Li, H. et al., 2015 [24]	7	147/3894	83,501/1,463,973	RR 1.20; 95% CI 1.02–1.42)		
Li, X. et al., 2015 [35]	4	117/2189	83,459/1,461,804	RR 1.29; 95% CI 1.07–1.55		

**Table 4:** Meta-analysis of risk ratios for congenital anomalies after exposure to both MMI/CMZ and PTU in pregnancy.

Author, Year	No. of Studies Included	MMI/CMZ + PTU	Control	Crude RR, 95% CI for Congenital Anomalies	Adjusted RR, 95% CI for Congenital Anomalies	No. of Anomalies/1000 Live Births
		Events/Total	Events/Total			
Agrawal et al., 2022 [33]	3	177/2126	254,049/4,318,697	RR 1.51; 95% CI, 1.14–1.99	RR 1.49; 95% CI, 1.20–1.84	31.4
Morales et al., 2021 [32]	3				RR 1.51, 95% CI 1.16–1.97	32.5
Song et al., 2017 [34]	2	30/285	83,333/1,446,588	RR 1.92; 95% CI, 1.32–2.81		
Li, H. et al., 2015 [24]	2	30/285	83,333/1,446,588	RR 1.83; 95% CI 1.30–2.56		
Li, X. et al., 2015 [35]	2	30/285	580/9475	RR 1.88; 95% CI 1.27–2.77		

**Table 5:** Medication used in the treatment of Graves’ disease and potential fetal risks.

Medication	Potential Fetal Risks
Propylthiouracil	cysts of the face and neck
	urinary abnormalities
Methimazole	aplasia cutis esophageal atresia choanal atresia abdominal wall defects
	cardiac (ventricular septal) defects
Radioactive iodine (I131)	fetal thyroid ablation
	malformations
	growth restriction
	mental retardation [48]
Beta-adrenergic blocking agents	intrauterine growth restriction
	fetal and neonatal bradycardia neonatal hypoglycemia

## Data Availability

Not applicable.

## References

[1] Aghajanian P. (2018). Preconception counseling for thyroid disorders. Ann. Thyroid..

[2] Alexander E.K., Pearce E.N., Brent G.A., Brown R.S., Chen H., Dosiou C., Grobman W.A., Laurberg P., Lazarus J.H., Mandel S.J. (2017). 2017 Guidelines of the American Thyroid Association for the Diagnosis and Management of Thyroid Disease During Pregnancy and the Postpartum. Thyroid.

[3] Kahaly G.J., Bartalena L., Hegedüs L., Leenhardt L., Poppe K., Pearce S.H. (2018). 2018 European Thyroid Association Guideline for the Management of Graves’ Hyperthyroidism. Eur. Thyroid. J..

[4] Korelitz J.J., McNally D.L., Masters M.N., Li S.X., Xu Y., Rivkees S.A., Alexander E.K., Mandel S.J., Prunty J.J., Heise C.D. (2013). Prevalence of Thyrotoxicosis, Antithyroid Medication Use, and Complications among Pregnant Women in the United States. Thyroid.

[5] Wang W., Teng W., Shan Z., Wang S., Li J., Zhu L., Zhou J., Mao J., Yu X., Li J. (2011). The prevalence of thyroid disorders during early pregnancy in China: The benefits of universal screening in the first trimester of pregnancy. Eur. J. Endocrinol..

[6] Cooper D.S., Laurberg P. (2013). Hyperthyroidism in pregnancy. Lancet Diabetes Endocrinol..

[7] Derakhshan A., Peeters R.P., Taylor P.N., Bliddal S., Carty D.M., Meems M., Vaidya B., Chen L., A Knight B., Ghafoor F. (2020). Association of maternal thyroid function with birthweight: A systematic review and individual-participant data meta-analysis. Lancet Diabetes Endocrinol..

[8] Ashkar C., Sztal-Mazer S., Topliss D.J. (2023). How to manage Graves’ disease in women of childbearing potential. Clin. Endocrinol..

[9] Korevaar T.I.M., Muetzel R., Medici M., Chaker L., Jaddoe V.W.V., de Rijke Y.B., Steegers E.A.P., Visser T.J., White T., Tiemeier H. (2016). Association of maternal thyroid function during early pregnancy with offspring IQ and brain morphology in childhood: A population-based prospective cohort study. Lancet Diabetes Endocrinol..

[10] Sreelatha S., Nadagoudar S., Devi A.L. (2017). The study of maternal and fetal outcome in pregnant women with thyroid disorders. Int. J. Reprod. Contraception, Obstet. Gynecol..

[11] Andersen S.L., Knøsgaard L., Olsen J., Vestergaard P., Andersen S. (2019). Maternal Thyroid Function, Use of Antithyroid Drugs in Early Pregnancy, and Birth Defects. J. Clin. Endocrinol. Metab..

[12] Mahadik K., Choudhary P., Roy P.K. (2020). Study of thyroid function in pregnancy, its feto-maternal outcome; a prospective observational study. BMC Pregnancy Childbirth.

[13] Panaitescu A.M. (2021). Pregnancy in Women with Graves’ Disease: Focus on Fetal Surveillance. Graves’ Disease.

[14] Goldman A.M., Mestman J.H. (2011). Transient non-autoimmune hyperthyroidism of early pregnancy. J. Thyroid. Res..

[15] Caron P. (2022). Management of thyrotoxicosis and pregnancy: Review of the current literature and an update of the care pathway. Ann. Endocrinol..

[16] Andersen S.L., Knøsgaard L. (2020). Management of thyrotoxicosis during pregnancy. Best Pract. Res. Clin. Endocrinol. Metab..

[17] Okosieme O.E., Khan I., Taylor P.N. (2018). Preconception management of thyroid dysfunction. Clin. Endocrinol..

[18] Iijima S. (2020). Pitfalls in the assessment of gestational transient thyrotoxicosis. Gynecol. Endocrinol..

[19] Pearce E.N. (2019). Management of thyrotoxicosis: Preconception, pregnancy, and the postpartum period. Endocr. Pract..

[20] Gaberšček S., Zaletel K. (2014). Thyroid physiology and autoimmunity in pregnancy and after delivery. Expert Rev. Clin. Immunol..

[21] Panaitescu A.M., Nicolaides K. (2017). Maternal autoimmune disorders and fetal defects. J. Matern. Neonatal Med..

[22] Quintino-Moro A., Zantut-Wittmann D.E., Tambascia M., MacHado H.D.C., Fernandes A. (2014). High Prevalence of Infertility among Women with Graves’ Disease and Hashimoto’s Thyroiditis. Int. J. Endocrinol..

[23] Nguyen C.T., Mestman J.H. (2019). Graves’ hyperthyroidism in pregnancy. Curr. Opin. Endocrinol. Diabetes Obes..

[24] Li H., Zheng J., Luo J., Zeng R., Feng N., Zhu N., Feng Q. (2015). Congenital Anomalies in Children Exposed to Antithyroid Drugs In-Utero: A Meta-Analysis of Cohort Studies. PLoS ONE.

[25] Luewan S., Chakkabut P., Tongsong T. (2011). Outcomes of pregnancy complicated with hyperthyroidism: A cohort study. Arch. Gynecol. Obstet..

[26] Smit B.J., Kok J.H., De Vries L.S., Vulsma T., Smolders-De Haas H., Briet J.M., Boer K., Wiersinga W.M. (1999). The Neurologic Development of the Newborn and Young Child in Relation to Maternal Thyroid Function. Pediatr. Res..

[27] Turunen S., Vääräsmäki M., Lahesmaa-Korpinen A., Leinonen M.K., Gissler M., Männistö T., Suvanto E. (2020). Maternal hyperthyroidism and pregnancy outcomes: A population-based cohort study. Clin. Endocrinol..

[28] Laurberg P., Wallin G., Tallstedt L., Abraham-Nordling M., Lundell G., Törring O. (2008). TSH-receptor autoimmunity in Graves’ disease after therapy with anti-thyroid drugs, surgery, or radioiodine: A 5-year prospective randomized study. Eur. J. Endocrinol..

[29] Seo G.H., Kim T.H., Chung J.H. (2018). Antithyroid drugs and congenital malformations a nationwide Korean cohort study. Ann Intern Med..

[30] Yoshihara A., Noh J., Yamaguchi T., Ohye H., Sato S., Sekiya K., Kosuga Y., Suzuki M., Matsumoto M., Kunii Y. (2012). Treatment of graves’ disease with antithyroid drugs in the first trimester of pregnancy and the prevalence of congenital malformation. J. Clin. Endocrinol. Metab..

[31] Chen C.-H., Xirasagar S., Lin C.-C., Wang L.-H., Kou Y., Lin H.-C. (2011). Risk of adverse perinatal outcomes with antithyroid treatment during pregnancy: A nationwide population-based study. BJOG Int. J. Obstet. Gynaecol..

[32] Morales D.R., Fonkwen L., Nordeng H.M.E. (2021). Antithyroid drug use during pregnancy and the risk of birth defects in offspring: Systematic review and meta-analysis of observational studies with methodological considerations. Br. J. Clin. Pharmacol..

[33] Agrawal M., Lewis S., Premawardhana L., Dayan C.M., Taylor P.N., Okosieme O.E. (2022). Antithyroid drug therapy in pregnancy and risk of congenital anomalies: Systematic review and meta-analysis. Clin. Endocrinol..

[34] Song R., Lin H., Chen Y., Zhang X., Feng W. (2017). Effects of methimazole and propylthiouracil exposure during pregnancy on the risk of neonatal congenital malformations: A meta-analysis. PLoS ONE.

[35] Li X., Liu G.Y., Ma J.L., Zhou L. (2015). Risk of congenital anomalies associated with antithyroid treatment during pregnancy: A me-ta-analysis. Clinics.

[36] Miao Y., Xu Y., Teng P., Wang A., Zhang Y., Zhou Y., Liu W. (2022). Efficacy of propylthiouracil in the treatment of pregnancy with hyperthyroidism and its effect on pregnancy outcomes: A meta-analysis. PLoS ONE.

[37] Tonacchera M., Chiovato L., Bartalena L., Cavaliere A.F., Vitti P. (2020). Treatment of Graves’ hyperthyroidism with thionamides: A position paper on indications and safety in pregnancy. J. Endocrinol. Investig..

[38] Yu W., Wu N., Li L., Wang J., OuYang H., Shen H. (2020). Side effects of PTU and MMI in the treatment of hyperthyroidism: A systematic review and meta-analysis. Endocr. Pract..

[39] Andersen S.L., Lönn S., Vestergaard P., Törring O. (2017). Birth defects after use of antithyroid drugs in early pregnancy: A Swedish nationwide study. Eur. J. Endocrinol..

[40] Sarkar S., Bischoff L.A. (2016). Management of Hyperthyroidism during the Preconception Phase, Pregnancy, and the Postpartum Period. Semin. Reprod. Med..

[41] Yoshihara A., Noh J.Y., Watanabe N., Mukasa K., Ohye H., Suzuki M., Matsumoto M., Kunii Y., Suzuki N., Kameda T. (2015). Substituting Potassium Iodide for Methimazole as the Treatment for Graves’ Disease During the First Trimester May Reduce the Incidence of Congenital Anomalies: A Retrospective Study at a Single Medical Institution in Japan. Thyroid.

[42] Alexander E.K., Larsen P.R. (2002). High Dose^131^I Therapy for the Treatment of Hyperthyroidism Caused by Graves’ Disease. J. Clin. Endocrinol. Metab..

[43] De Jong J.A.F., Verkooijen H.M., Valk G.D., Zelissen P.M.J., De Keizer B. (2013). High failure rates after 131I therapy in graves hyperthy-roidism patients with large thyroid volumes, high iodine uptake, and high iodine turnover. Clin. Nucl. Med..

[44] Moura-Neto A., Mosci C., Santos A.O., Amorim B.J., de Lima M.C.L., Etchebehere E.C.S.C., Tambascia M.A., Ramos C.D., Zantut-Wittmann D.E. (2012). Predictive factors of failure in a fixed 15 mCi 131I-Iodide therapy for Graves’ Disease. Clin. Nucl. Med..

[45] Schneider D.F., Ba P.E.S., Bs M.F.J., Ojomo K.A., Chen H., Jaume J.C., Elson D.F., Perlman S.B., Sippel R.S. (2014). Failure of Radioactive Iodine in the Treatment of Hyperthyroidism. Ann. Surg. Oncol..

[46] Laurberg P., Bournaud C., Karmisholt J., Orgiazzi J. (2009). Management of Graves’ hyperthyroidism in pregnancy: Focus on both maternal and foetal thyroid function, and caution against surgical thyroidectomy in pregnancy. Eur. J. Endocrinol..

[47] Sherif I.H., Oyan W.T., Bosairi S., Carrascal S.M. (1991). Treatment of hyperthyroidism in pregnancy. Acta Obstet. Gynecol. Scand..

[48] Hyer S., Pratt B., Newbold K., Hamer C. (2011). Outcome of Pregnancy after Exposure to Radioiodine in Utero. Endocr. Pract..

[49] Rochester D.B., Davies T.F. (2005). Increased risk of Graves’ disease after pregnancy. Thyroid.

[50] Tagami T., Hagiwara H., Kimura T., Usui T., Shimatsu A., Naruse M., Prunty J.J., Heise C.D., Chaffin D.G., Yalamanchi S. (2007). The Incidence of gestational hyperthyroidism and postpartum thyroiditis in treated patients with Graves’ disease. Thyroid.

